# Verbal and non-verbal recognition memory assessment: validation of a computerized version of the Recognition Memory Test

**DOI:** 10.1007/s10072-023-07171-3

**Published:** 2023-12-22

**Authors:** Elena Baruzzo, Stefano Terruzzi, Beatrice Feder, Costanza Papagno, Daniela Smirni

**Affiliations:** 1 Center for Mind/Brain Sciences-CIMeC, University of Trento, Rovereto, Italy; 2 Department of Psychology, Educational Science and Human Movement, University of Palermo, Palermo, Italy

**Keywords:** Recognition Memory Test, RMT, Neuropsychological assessment, Computerized assessment, Digital assessment

## Abstract

**Background:**

The use of computerized devices for neuropsychological assessment (CNADs) as an effective alternative to the traditional pencil-and-paper modality has recently increased exponentially, both in clinical practice and research, especially due to the pandemic. However, several authors underline that the computerized modality requires the same psychometric validity as "in-presence" tests. The current study aimed at building and validating a computerized version of the verbal and non-verbal recognition memory test (RMT) for words, unknown faces and buildings.

**Methods:**

Seventy-two healthy Italian participants, with medium–high education and ability to proficiently use computerized systems, were enrolled. The sample was subdivided into six groups, one for each age decade. Twelve neurological patients with mixed aetiology, age and educational level were also recruited. Both the computerized and the paper-and-pencil versions of the RMT were administered in two separate sessions.

**Results:**

In healthy participants, the computerized and the paper-and-pencil versions of the RMT showed statistical equivalence for words, unknown faces and buildings. In the neurological patients, no statistical difference was found between the performance at the two versions of the RMT. A moderate-to-good inter-rater reliability between the two versions was also found in both samples. Finally, the computerized version of the RMT was perceived as acceptable by both healthy participants and neurological patients at System Usability Scale (SUS).

**Conclusion:**

The computerized version of the RMT can be used as a reliable alternative to the traditional version.

## Introduction

In the past few decades, the use of computerized neuropsychological assessment devices (CNADs) has received increasing attention in both clinical and research settings, because it is considered as a more advantageous alternative to conventional examiner-based tests [1]. The interest in CNADs has exponentially increased in response to the COVID-19 pandemic, which has forced significant changes in clinical practice. Indeed, one of the properties of CNADs is to allow remote encounters between clinicians and patients [see 2 for review]. Furthermore, focusing on the fact that neuropsychological tests on digital platforms are sensitive and cost-effective, several studies have already used these tools with many clinical populations [3]. However, to date, "paper-and-pencil" tests remain the gold standard for neuropsychological assessment [4, 5]. The American Academy of Clinical Neuropsychology and the National Academy of Neuropsychology, in a joint document, defined CNADs as any tool that administers, evaluates, or interprets tests of brain function using a computer, portable devices (such as digital tablets or smartphones), or any other digital interface instead of a human examiner [3]. However, such a position statement, that emphasizes the administration of cognitive tests by a computer "*instead of*" a human examiner, can be extended to all computerized neuropsychological platforms, including a broader spectrum of ever-growing technology and, therefore, include tools that may allow a better presentation of the stimuli and data recording through computer presentation (such as immersive and non-immersive Virtual Reality) [4]. As aforementioned, CNADs allow to present with extreme accuracy a wider range of tasks including, for example, those involving multi-tasking, multiple measurements, divided attention, speed processing and response times for multiple measurements. Furthermore, CNADs provide new parameters, more easily and quickly available [as, for instance, recording of response latencies, see 6], leading to greater accuracy in detecting cognitive changes [7–9], in both the clinical and research fields [10, 11]. Finally, computerized tests can be administered to many subjects in shorter times and with lower costs; the evaluation of the responses can be more precise, enabling automatic administration and scoring procedures, and thus reducing the probability of human error [3]. On the other hand, in a conventional neuropsychological assessment, there can be a great deal of variation between different examiners or between the same examiner along an assessment session, in the manual use of tools (such as a stopwatch), or in the manual presentation of material (such as stimulus cards) [12]. Furthermore, while in an examiner-centred paper-and-pencil assessment the patient interacts with an examiner who presents stimuli, records responses, notes key behavioral observations and also understands the patient’s level of effort and motivation, in CNADs, the patient interacts with a computerized test station or a tablet through one or more alternative input/output devices (keyboard, voice, mouse, joystick, touchscreen, head-mounted display), sometimes without any supervision or observation. Additionally, computerized assessment does not permit examiners to introduce flexibility into their evaluation or provide any structured encouragements to the examinee. For those reasons, the use of CNADs may require familiarity with technical skills that can significantly impact on relevant evaluation parameters. Patients, especially the elderly, may have limited familiarity with computer interfaces, as well as negative attitudes and anxiety about computers [13]: as a result, their performance may differ significantly from pencil-and-paper measurements [14–16]. On these assumptions, computerized tests are not simply the replacement of paper-and-pencil for a computer screen and electronic response capture and they could not be considered *tout court* directly comparable to an examiner-administered evaluation [17–19]. Rather, a traditional test administered by computer becomes a new test, different from the conventional one both qualitatively and technically. Indeed, several studies have proved that there are substantial differences between the computerized measurements and the corresponding examiner administered tests in different samples [20–23]. In addition, the "replication crisis" [24] has increased the need for appropriate and robust statistical inference: when examiner-based tests are proposed in a computerized version, new psychometric data should be provided according to the same standards of psychometric tests. It cannot be assumed a priori that the normative data for a test administered by the examiner apply equally well to a computerized version of the same test, due to changes in the administration method and patients’ familiarity with the computer. Thus, if a computerized test adapted from a conventional test is not simply a slightly different format for an existing test, it becomes essential that equivalence testing between the paper-and-pencil and the computerized versions of that test is provided. New normative data for computerized tests must be collected when no equivalence with the paper-and-pencil version of the tests is proved. Several tests have already been adapted for computerized presentation, such as the Montreal Cognitive Assessment – MoCA [25], the Clock Drawing Test, the Pentagon Drawing Test [26], the Rey Complex Figure copy task [27], the Bells Cancellation, Line Bisection and Five Elements Drawing Tests [28]: these tests are, of course, critical in the assessment of cognitive functioning.

Among neuropsychological patients, memory problems are one of the most reported symptoms and, due to growing life expectancy, differentiating early neurodegenerative disorders from normal aging is for sure one of the greatest challenges for the future. Notably, despite the extensive body of literature supporting the use of recognition memory paradigms to enhance differential diagnosis between neurological patients, depressed ones and healthy controls [29], its clinical assessment is still limited due to the paucity of validated, clinical tools available. As for the Italian population, only the verbal and non-verbal recognition memory test (RMT) [30] and the visual long-term recognition memory test [31] are available. The RMT represents a relatively fast screening tool, which may be useful for the early detection of memory disorders; two parallel versions are also available for multiple testing [32]. However, despite its promising features, the original examiner-centred version of the RMT may be prone to human error: clinicians are indeed required to control for several factors, namely the duration of the stimuli presentation and the registration of patients’ responses and behaviours [see 30, methods section]. For all these reasons, we decided to provide a validation of a computerized version of the verbal and non-verbal recognition memory test (RMT—Form A) for words, unknown faces, and buildings [32], comparing it to the results of the original paper-and-pencil test. To this end, a computerized version of the RMT was built and we administered both the pencil-and-paper and the computerized version to different age-balanced groups of healthy participants, with medium–high education. Furthermore, in order to evaluate the possible impact of different neurological conditions on patients' ability to interact with the computer interface, the two version of the RMT were also administered to a small sample of neurological patients with mixed aetiology.

## Materials and methods

### Materials

The previously published battery of verbal and non-verbal recognition memory tests (RMT) [32] and its computerized version were used. The battery evaluates different components of recognition memory for words, unfamiliar faces, and unknown buildings. Stimuli selection and test construction are described elsewhere [30]. Briefly, the original, pencil-and-paper version had the same administration procedure for the three subtasks (*i.e.*, for words, faces and buildings): during the study phase, the 30 target stimuli were individually displayed at a 3-s. interval per item and, for each stimulus, participants were asked to judge whether they like it or not to enhance an adequate level of attention. In the recognition phase, performed immediately after the end of the study phase, three stimuli were presented simultaneously: the target stimulus and two distractors. Participants were asked to recognize, among the three items, the previously seen one. Targets were distributed randomly to the top, bottom, or centre of the sheet, ensuring an equal number of placements in the three positions. The number of correct answers corresponds to the total score of each subtask (*range: 0–30*). Overall, the test lasts about 20 min.

**Fig. 1:**
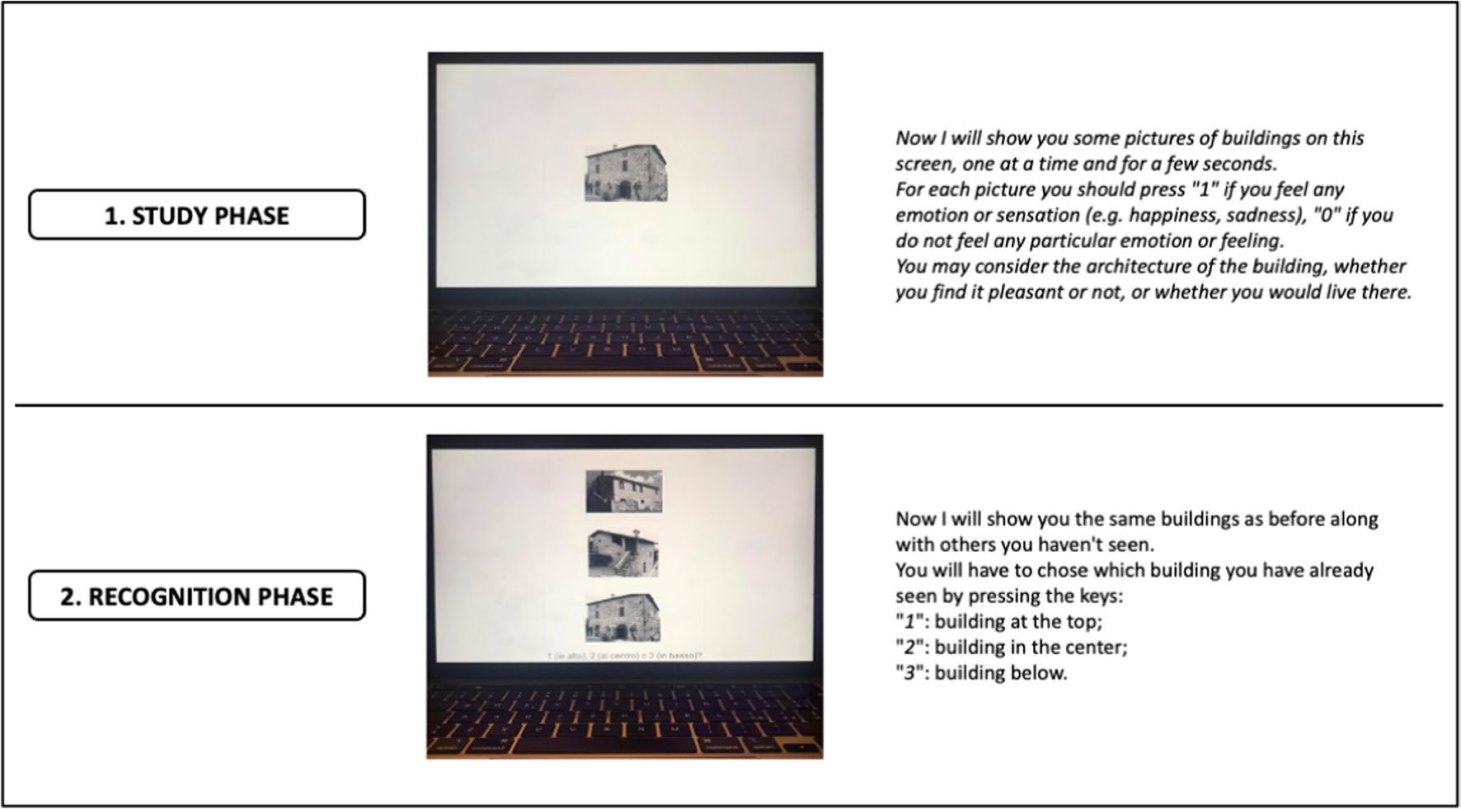
Example of the study and recognition phases for the computerized version of the RMT

Here we present a computerized version of the RMT. The test was built on PsychoPy and the stimuli were shown to the participants using a MacBook Pro 13" (with a set screen resolution of 1680 × 1050). The stimuli were the same as in Smirni et al.’s [30] original version and, in the study phase, each stimulus was displayed at a 3-s. interval. To ensure an adequate level of attention on each stimulus, participants were asked to press "1" on the keyboard if it produced any emotion or feeling (e.g. happiness, sadness, etc.), "0" if it did not produce any particular emotion or feeling (Fig. 1). With respect to the original one, in this computerized version, administration and scoring procedures are fully automatic: indeed, instructions are shown on the PC/laptop monitor and participants are invited to provide responses using some keys on the keyboard. Furthermore, after each subtest completion, an electronic sheet with all the answers provided by the participant, the raw score and its conversion into adjusted and equivalent scores is available. The system automatically applies the regression formulas of Smirni et al. [30] to the raw scores obtained by the participants, even in the case of minimum or maximum raw score (0/30 and 30/30 respectively). In agreement with Novelli et al. [33], in the former case the equivalent score is always zero, in the latter, the equivalent score is equal to four, disrespectfully from the foreseen automatic correction.

In addition, the participants were administered the Montreal Cognitive Assessment (MoCA) [34] to assess the general cognitive level, and the System Usability Scale (SUS) [35], which evaluates the subjective opinion from a user regarding a particular software. The SUS consists of 10 items in which even-numbered questions are posed in negative form. All the items need to be rated on a Likert scale ranging from 1 (completely disagree), to 5 (completely agree).

### _Methods_

The two versions of the RMT (*i.e.*, the original pencil-and-paper and the computerized versions) were administered by one of the authors (BF) to a sample of 72 healthy participants, in two testing sessions over two separate days, at least one week apart and within 10 days. The examiner was always present in the same room during the computerized administration of the test, but no further instructions or clarifications were requested from the examinees. The administration order of the two versions (first pencil-and-paper; first computerized), was counterbalanced across subjects as well as the administration order of the three subtasks. Inclusion criteria for participants were: i) age between 20 and 79 years; ii) at least 5 years of education. Exclusion criteria were: i) a history or clinical evidence of neurological or neuropsychiatric disease; ii) subjective complaint of memory or other cognitive impairments; iii) a Montreal Cognitive Assessment (MoCA) adjusted score below the Italian cut-off score [34]. The sample was then subdivided into six groups, one for each age decade; the number of males and females was matched across the six groups.

Furthermore, a continuous series of 12 neurological patients with mixed aetiology, age and educational level was recruited at the Centre for Cognitive Rehabilitation (CeRiN) between May and August 2023. The two versions of the test RMT were administered by two psychologists expert in neuropsychology (EB and ST), to evaluate the possible impact of different neurological conditions on patients' ability to interact with the computer interface. The administration order of the two versions was counterbalanced across the patients as well as the administration order of the three subtasks.

Written informed consent was obtained from all participants and the study was conducted in accordance with the Declaration of Helsinki, with the approval of the local ethics committee.

Socio-demographic characteristics of both healthy participants and neurological patients are shown in Table 1, together with the SUS scores.

**Table 1:** Socio-demographic characteristics, MoCA’s adjusted score and SUS’s raw score from the healthy participants (top of the table), and the neurological patients (bottom of the table)

Healthy participants						
Decade	*N*	*M/F*	*Age (years* ± *sd)*	*Education (years* ± *sd)*	*MoCA (adjusted score* ± *sd)*	*SUS (raw score* ± *sd)*
20–29	12	6/6	26.60 (1.63)	17.25 (1.14)	26.03 (2.52)	96.46 (5.48)
30–39	12	6/6	33.06 (2.43)	15.50 (2.39)	26.60 (2.06)	95.00 (8.05)
40–49	12	6/6	44.09 (2.32)	15.58 (3.99)	27.10 (2.53)	93.96 (9.62)
50–59	12	6/6	56.99 (2.19)	11.67 (2.67)	26.75 (1.68)	98.54 (1.98)
60–69	12	6/6	64.58 (3.05)	13.92 (3.75)	25.92 (1.25)	93.75 (5.69)
70–79	12	6/6	74.47 (2.66)	10.33 (3.42)	25.12 (2.25)	96.67 (2.89)
Total	72	36/36	49.97 (17.26)	14.04 (3.80)	26.25 (2.13)	97.52 (6.23)
Neurological patients						
ID	*Aetiology*	*Sex*	*Age*	*Education*	*MoCA (adjusted score)*	*SUS (raw score)*
1	Traumatic brain injury	M	75	8	19.837	92.5
2	Amnesic Mild Cognitive Impairment	M	76	13	19.668	92.5
3	Haemorrhagic stroke	M	31	13	19.168	100
4	Ischemic stroke	M	66	13	12.918	57.5
5	Posterior cortical atrophy	F	52	13	11.168	87.5
6	Ischemic stroke	F	31	18	22.729	75
7	Brain tumour	M	20	11	23.337	92.5
8	Ischemic stroke	F	29	18	22.168	92.5
9	Ischemic stroke	F	59	11	20.216	92.5
10	Ischemic stroke	M	68	8	18.087	87.5
11	Body Lewy dementia	M	71	18	18.354	57.5
12	Multi-domain Mild Cognitive Impairment	M	68	17	21.354	85
Total	-	8 M/4F	50.83 (20.48)	13.41 (3.65)	19.08 (3.69)	84.4. (13.9)

*N* number; *M* males; *F* females; *MoCA* Montreal Cognitive Assessment; *SUS* System Usability Scale

## Statistical analysis

### Learning effect

A series of linear mixed effects models were run to check for possible effects of learning. The raw scores at the three RMT subtasks were considered as a continuous dependent variable. As a factorial variable, the version of test (Version, two levels: paper-and-pencil; computerized), the administration order of the two versions (Version order, two levels: first paper-and-pencil; first computerized), and the administration order of the three subtasks (Task order; six levels: faces-words-buildings; faces-buildings-words; buildings-faces-words; buildings-words-faces; words-faces-buildings; words-buildings-faces), were added to the model. A by subject random intercept was also added to account for inter-subject variability. The same analysis was separately performed for healthy participants and neurological patients.

### Inter-rater reliability

Inter-rater reliability between the computerized and the paper-and-pencil versions of the RMT was separately assessed for healthy participants and neurological patients through intra-class correlations. Results were interpreted according to Koo and Li’s guidelines [36].

### Healthy participants

Equivalence between the two versions of the RMT for faces, buildings and words was assessed through a two one-sided test procedure (TOST) for dependent samples [37]. Indeed, while the lack of a significant difference between scores on a paired *t* test cannot be taken as an evidence of agreement, equivalence tests demonstrate that scores from different tests are equal within a predetermined boundary. Normality assumptions on raw variables were checked by the evaluation of skewness and kurtosis values (judged as abnormal if ≥|1| and |3|, respectively [38]). Given the non-normality of the raw scores, data were reflected and log transformed prior to analyses with equivalence tests. The TOST procedure involves an a priori determination of an equivalence boundary (± delta), within which score difference can be considered trivial. Delta was set a priori at 1.1 raw score points, which log transforms to 0.1 and results in an equivalence interval of − 0.1 and 0.1. α level was set at 0.005.

### Neurological patients

The presence of any difference in the performance between the two versions of the RMT for faces, buildings and words was assessed through paired samples t-tests.

## Results

### Learning effect

When testing for possible effects of learning in healthy participants, ANOVA on the run final models did not showed a significant 3-way interaction effect (Version*Version order*Task order) for any of the three RMT subtasks (words: F_(5, 60)_ = 1.102, *p* = 0.369; faces: F_(5, 60)_ = 0.154, *p* = 0.987; buildings: F_(5, 60)_ = 0.543, *p* = 0.743).

Similarly, no interaction effect (Version*Version order*Task order), was found when checking for a possible learning effect in the neurological patients for any of the three RMT subtasks (words: F_(3,2)_ = 3.379, *p* = 0.237; faces: F_(3,2)_ = 8.063, *p* = 0.112; buildings: F_(3,2)_ = 0.573, *p* = 0.686).

### Inter-rater reliability

In the healthy participants, inter-rater reliability between the two versions of the RMT was moderate for faces (ICC = 0.699), buildings (ICC = 0.644) and words (ICC = 0.746). In the neurological patients inter-rater reliability was moderate for buildings (ICC = 0.539), and good for faces (ICC = 0.756) and words (ICC = 0.836).

### Healthy participants

**Fig. 2:**
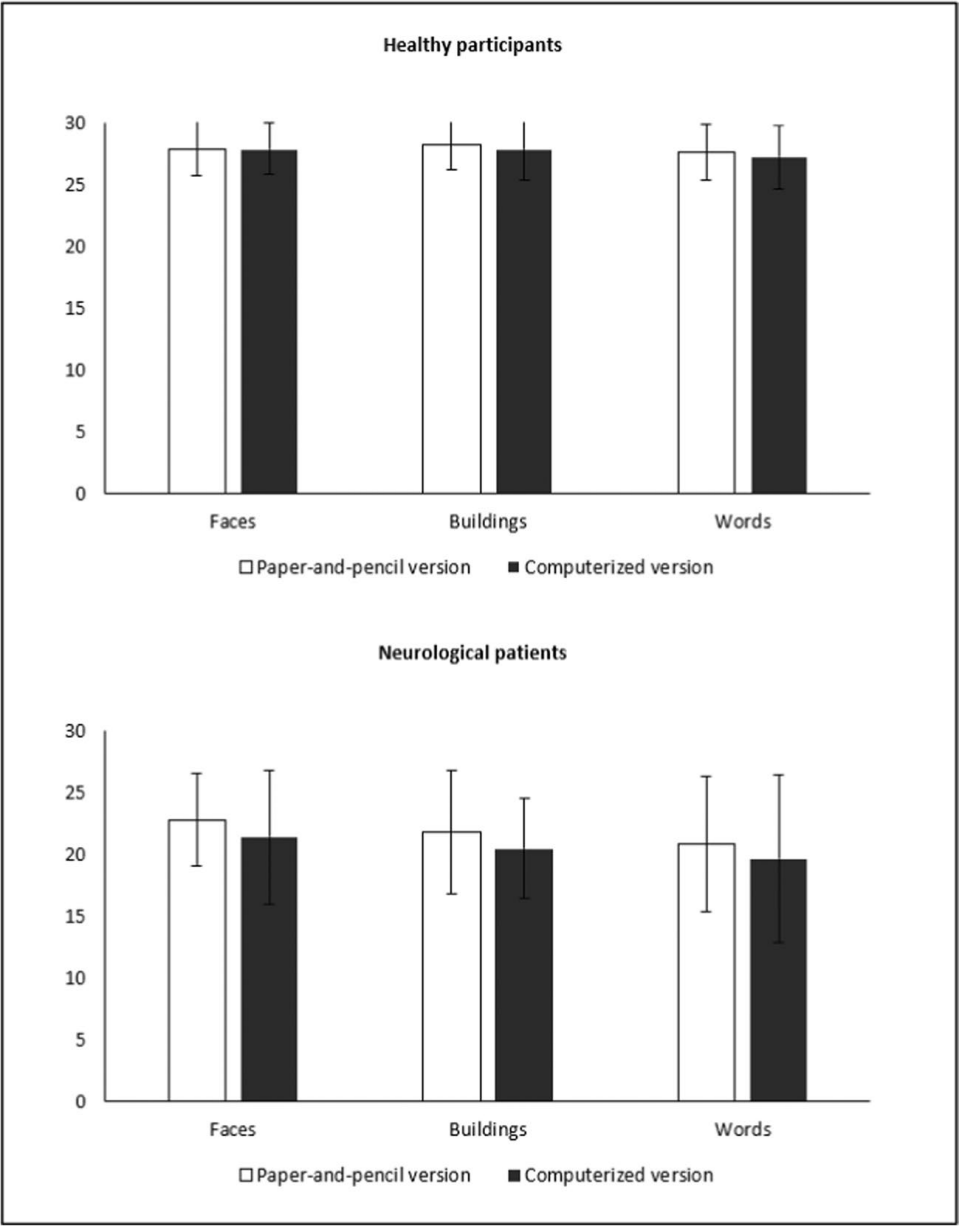
Mean and standard error of the scores obtained by the healthy participants (top of the image), and the neurological patients (bottom of the image), in the three subtasks of the two version of the RMT

When comparing the two versions of the RMT for faces, no significant differences were found (t_(71)_ = 0.298, *p* = 0.767, d = 0.003). Consistently, they also showed statistical equivalence on the TOST procedure (both upper and lower equivalence bound yielding a *p* < 0.005; computerized version mean = 27.95 ± 2.11; paper-and-pencil version mean = 27.93 ± 2.20). Also when comparing the two versions of the RMT for buildings, no significant effect was found (t_(71)_ = 895, *p* = 0.374, d = 0.105). Consistently, they also showed statistical equivalence on the TOST procedure (both upper and lower equivalence bound yielding a *p* < 0.005; computerized version mean = 27.87 ± 2.46; paper-and-pencil version mean = 28.23 ± 1.98). Finally, no significant differences were found even when comparing the two versions of the RMT for words (t_(71)_ = 1.88, *p* = 0.064, d = 0.221). Consistently, they also showed statistical equivalence on the TOST procedure (both upper and lower equivalence bound yielding a *p* < 0.005; computerized version mean = 27.25 ± 2.56; paper-and-pencil version mean = 27.66 ± 2.26) (Fig. 2). Table 2 reports means and standard deviations scores obtained by the sample at the two versions of the RMT (paper-and-pencil, computerized), in all the three subtests (words, unfamiliar faces and unknown buildings), for each decade of age.

**Table 2:** Mean scores and standard deviations of the two versions of the RMT in the validation sample divided by decades of age (top of the table), and in the neurological patients (bottom of the table)

Healthy participants						
Version →	*Paper-and-pencil*			*Computerized*		
Decade ↓	*Faces*	*Words*	*Buildings*	*Faces*	*Words*	*Buildings*
20–29	28.75 (1.54)	29.42 (.99)	28.92 (1.08)	29.08 (.99)	29.17 (.71)	28.83 (1.80)
30–39	28.00 (2.17)	28.08 (1.31)	28.58 (1.37)	27.42 (2.06)	27.25 (2.13)	28.58 (1.44)
40–49	28.08 (2.10)	27.92 (2.15)	29.08 (.90)	28.08 (1.92)	27.42 (3.05)	28.58 (1.24)
50–59	28.17 (2.32)	27.17 (2.69)	28.50 (1.78)	28.83 (.93)	26.67 (3.14)	28.75 (1.05)
60–69	27.92 (2.35)	26.42 (2.46)	27.92 (1.31)	27.92 (2.71)	25.83 (2.72)	27.17 (2.28)
70–79	26.73 (2.61)	26.92 (2.50)	26.42 (3.39)	26.42(2.57)	26.08 (2.87)	25.33 (3.91)
*Total*	27.93 (2.20)	27.62 (2.26)	28.23 (1.98)	27.95 (2.11)	27.06 (2.72)	27.87 (2.46)
Neurological patients						
Version →	*Paper-and-pencil*			*Computerized*		
Mean (sd)	22.83 (3.77)	20.91 (5.50)	21.83 (4.96)	21.41 (5.44)	19.66 (6.80)	20.50 (4.10)

### Neurological patients

When comparing the two version of the RMT, no significant differences were found for faces (computerized version mean = 21.41 ± 5.44; paper-and-pencil version mean = 22.83 ± 3.79; t_(11)_ = 1.48, *p* = 0.167, d = 0.427), buildings (computerized version mean = 20.50 ± 4.10; paper-and-pencil version mean = 21.83 ± 4.96; t_(11)_ = 1.07, *p* = 0.309, d = 0.308), or words (computerized version mean = 19.66 ± 6.80; paper-and-pencil version mean = 20.91 ± 5.50; t_(11)_ = 1.29, *p* = 0.224, d = 0.372). (Fig. 2). Table 2 reports means and standard deviations scores obtained by the neurological patients at the two versions of the RMT.

### System usability scale – SUS

**Fig. 3:**
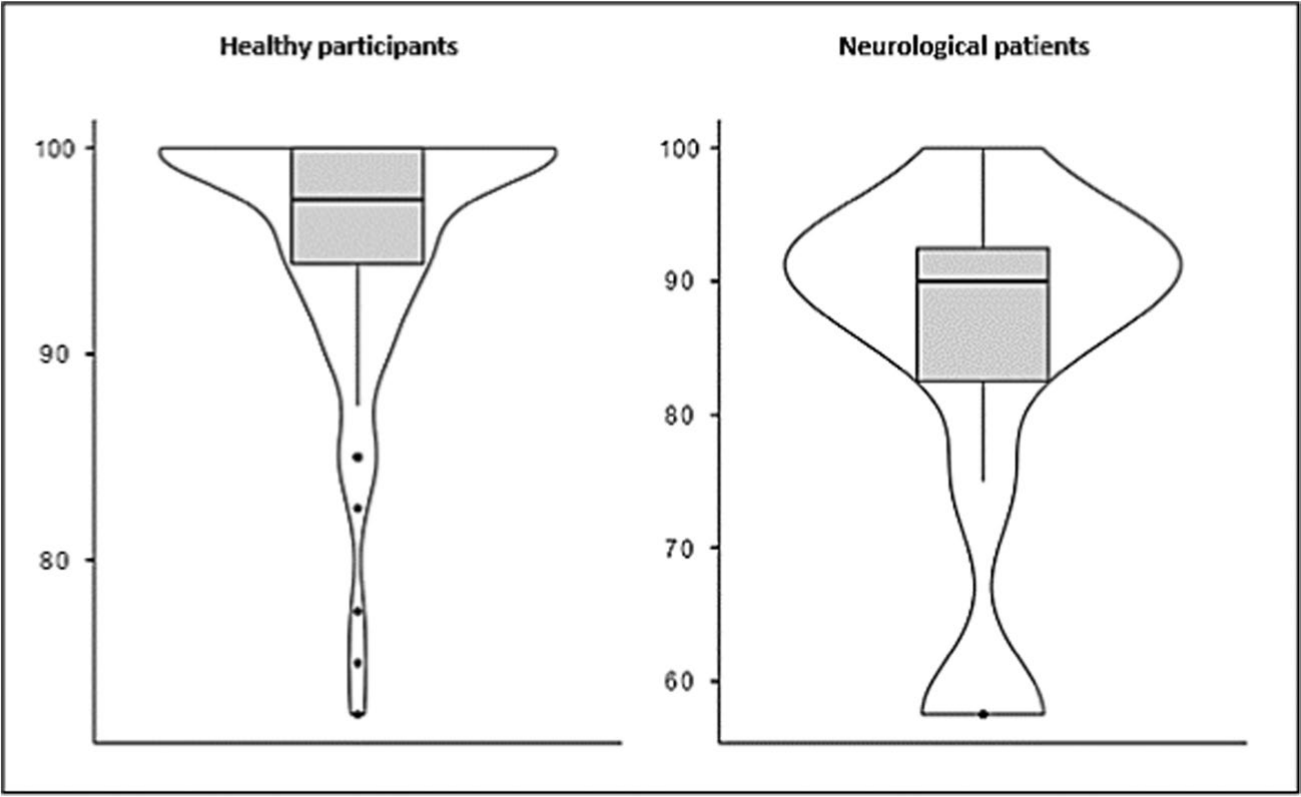
Distribution of the SUS scores from the healthy participants (on the left), and the neurological patients (on the right). Horizontal solid lines represent median and non-solid lines interquartile range

The SUS total median value from healthy participants was 97.5 (IQR = 5.63), with a minimum rating score of 72.5 and a maximum rating score of 100 (Fig. 3). The mean score of 95.7 (± 6.23), shows that the system is perceived as "excellent" and "acceptable" according to the adjective scale developed by Bangor et al. [39]. When evaluating the user experience of the neurological patients, the SUS total median value was 90.0 (IQR = 10.0), with a minimum rating score of 57.5 and a maximum rating score of 100 (Fig. 3). The mean score of 84.4 (± 13.9), shows that the system is perceived as "good" and "acceptable" [39].

## Discussion

In recent years, a large body of literature has shown an increasing interest in the use of computerized modalities in neuropsychological assessment both in clinical practice and in research [3, 7, 8, 12, 40]. CNADs have been proved to be highly effective, compared to the traditional assessment. On the other hand, this considerable enthusiasm is reduced by others studies arguing that the computerized tests cannot be considered *tout court* the equivalent of the conventional pencil-and-paper test [17, 21]. The same studies underlined that a computerized version requires the same psychometric validity of the tests directly administered by an examiner. In addition, these studies pointed out that the use of CNADs creates a specific evaluation setting in which the patient’s performance can be conditioned by the peculiar modality and then it may provide information that do not correspond to the real subject’s ability [17, 21].

According to this theoretical-applicative context, the present study aimed to create and validate a computerized version of the pencil-and-paper verbal and non-verbal recognition memory test (RMT—Form A) for words, unfamiliar faces and unknown buildings [32]. Both the pencil-and-paper and computerized versions were administered to different age-balanced groups of healthy participants, with medium–high education (mean education: 14.04 years; range: 8–25), and ability to use a high computerized system, proficiently and without performance slowdowns (SUS mean score: 97.52/100; range: 72.5–100). Our results showed a moderate inter-rater reliability between the two versions of the RMT for words, unknown faces and buildings. Furthermore, when comparing the two versions, no significant differences were found, and according to the TOST procedure, the three tests can be considered equivalent in both modalities (*i.e.*, computerized and paper-and-pencil). We also enrolled a small sample of neurological patients with mixed aetiology, age (range: 20–76) and educational level (range: 8–18) to evaluate the possible impact of different neurological conditions on the patients’ ability to interact with the computerized version of the RMT. Results showed a moderate-to-good inter-rater reliability between the two versions of the RMT. Furthermore, no difference was found in patient’s performance between the two versions of the RMT for words, unknown faces and buildings. Finally, results on SUS showed that the system is perceived as "good" and "acceptable" [39] (SUS mean score: 84.4/100; range: 57.5–100). Moreover, none of the patients referred feeling of discomfort while interacting with the computer interface, nor did they need further instructions from the experimenter, others then those provided by the computerized test. These findings support the possibility to use, in clinical practice, the computerized version as an alternative to the traditional one.

The computerized version of the RMT can offer several advantages in the assessment procedures, such as, for example, the reduction of the examiner’s influence, higher consistency administration, presentation of the stimuli, and scoring. It can also support a higher number of screening and follow-up activities on large populations [7, 40, 41], due to the ease of data collection and the scoring procedure, the immediate availability of results and a significant reduction in costs linked to administering the tests. Furthermore, another strength is that, in the spirit of Open Science, test materials and instructions are freely available on the web under a Creative Common licence (https://osf.io/yuqbh/?view_only=1edfc3db31b74ac581f83dfbc4b7cced). Therefore, interested neuropsychologists can use them in both clinical and research settings. Finally, this computerized version of the RMT may be suitable for online assessment, which has recently been acknowledge as a reliable alternative to the "*in-presence*", traditional, neuropsychological assessment for diagnosing neurocognitive and degenerative disorders [42, 43], such as preclinical dementia [44], as well as in healthy population assessment and in Alzheimer’s disease [45]. Online assessment makes it certainly easier for patients to access medical care permitting to save time and money even if several potential limitations must be considered (e.g., technological devices malfunctioning during the session; video or audio quality variability during the assessment).

The computerized version of the RMT is not exempt from limitations. For example, this test cannot be administered to poorly educated people over 80 years, because they usually are not familiar with technological devices. In fact, our validation sample included participants aged from 20 to 79 years, with medium–high education, and their rating of usability for the computerized version of the RMT was almost at ceiling. However, technological devices are expected to become usual tools for the whole population.

## Data Availability

Datasets associated with the current study are available from the corresponding author upon request.
